# The influence of training load and schedule on youth athletes' sleep

**DOI:** 10.1111/jsr.70013

**Published:** 2025-02-17

**Authors:** Tanisha Tate, Spencer Roberts, Luana C. Main, Lyndell Bruce

**Affiliations:** ^1^ Deakin University Centre for Sport Research within the Institute for Physical Activity and Nutrition, School of Exercise and Nutrition Sciences Burwood Victoria Australia; ^2^ Deakin University Institute for Physical Activity and Nutrition, School of Exercise and Nutrition Sciences Burwood Victoria Australia

**Keywords:** adolescent, basketball, exercise, recovery, team sport

## Abstract

Sleep is important for youth athletes, supporting sport‐related recovery and performance, as well as growth and development. Sleep may be influenced by training factors; therefore, this study aimed to characterise youth athletes' sleep, and examine associations between training load, schedule and frequency, and sleep. Twenty‐six youth basketballers (age: 14.22 ± 0.74 years, 13 male, 13 female) from a high‐performance sporting school were monitored for a period of 8 weeks. Sleep measures (via actigraphy and sleep diaries) were collected alongside training diaries (recording time, duration and perceived exertion). Youth athletes who finished training after 20:30 hours had significantly less total sleep time than when training finished before 20:30 hours. Higher daily total training duration was associated with decreased total sleep time. There is a potential contradiction with findings related to the number of sessions per day, as participants who completed two training sessions in a day had more sleep than those who completed one session per day; but this was not observed for athletes with three sessions per day. Participants had large intra‐individual variations (mean intra‐individual standard deviation) in bedtime (1.06 hr) and sleep time (0.84 hr). In conclusion: (1) late night training sessions appear to reduce total sleep time and should be avoided in youth athletes; (2) total daily training duration had a greater negative effect on sleep than session frequency; and (3) participants’ large intra‐individual variation in bedtime may reduce sleep quality and efficiency.

## 1 INTRODUCTION

Sleep is a fundamental part of human function, with reductions in sleep quality and quantity negatively affecting immune function (Mejri et al., 2014), memory (Walker, 2008) and cognitive function (Sufrinko et al., 2016). Adequate sleep, which includes achieving the recommended sleep time and efficiency, in addition to good sleep habits is particularly critical for athletes to aid their recovery and optimise sport performance (Fullagar et al., 2015). Furthermore, within youth athletes, sleep is crucial for their growth and development alongside their sporting endeavours (Beebe, 2011). According to the National Sleep Foundation in the USA, the recommended guidelines for sleep for adolescents is 8–10 hr (Hirshkowitz et al., 2015). However, the opportunities for adequate sleep in youth athletes may be reduced due to the time commitments for training and games, as well as juggling other responsibilities like schooling, casual work and interpersonal relationships.

Previous research has shown that youth athletes are often unable to achieve, on average, more than 8 hr of sleep as recommended, and this is consistent for both genders (Lastella et al., 2020; Steenekamp et al., 2021). National youth female basketball players only obtained an average of 7.2–7.6 hr of sleep on training days (Lastella et al., 2020), whilst in club level youth swimming and rowing, athletes (male and female) achieved an average sleep duration of 7 hr and 55 min (Steenekamp et al., 2021). In addition, research has suggested that youth athletes have greater intra‐individual variability in their sleep when compared with the general adolescent population (Leduc et al., 2020). These studies identified that the lack of and variability of sleep could be caused by training factors. Training factors can include training frequency, timing and load, with load referring to either the duration or intensity of the session or a combination of both. Training load can be measured objectively via devices such as global positioning systems (GPS) and heart rate monitors (Halson, 2014); however, most research has used a subjective measure of training load, such as session rating of perceived exertion (sRPE), to examine its relationship with sleep (Aloulou et al., 2021; Frytz et al., 2023; Lastella et al., 2020; Whitworth‐Turner et al., 2019). The total sleep time of national youth female basketball athletes was significantly higher on rest days when compared with low, moderate and high training load days where training load was monitored using sRPE (Lastella et al., 2020). In youth swimming and rowing, the median sleep duration of athletes (male and female) on nights before early morning trainings was significantly shorter compared with when they had the day off (Steenekamp et al., 2021). This was due to athletes needing to wake earlier for training, but they were unable to adjust their bedtime the night before, therefore their total sleep time was shorter (Steenekamp et al., 2021). Although these studies investigated the impact of training load (Lastella et al., 2020) or training schedules (Steenekamp et al., 2021) on the sleep of youth athletes, only one study to date has collectively looked at the impact of training load, schedule and frequency on the sleep of youth athletes (Frytz et al., 2023). However, this study did not include training sessions that finished after 17:00 hours, and therefore may not be representative of many youth athletes that need to train later in the evening after school commitments.

Previous research has identified that the training of youth athletes may impact other aspects of their sleep, including sleep quality, sleep fragmentation, wake‐up time and sleep efficiency. National and international youth athletes with higher training loads (monitored by sRPE) had poorer sleep quality and an increase in sleep fragmentation, implying sleep disruption (Aloulou et al., 2021). Whitworth‐Turner et al. (2019) also identified that sleep can be sensitive to training load in youth soccer academy athletes, noting that for every 100 arbitrary unit (au) increase in sRPE, this resulted in time of awakening being on average 4 min later. When youth female basketball athletes had low load training days as measured by sRPE (i.e. ~274 au), they had a higher sleep efficiency compared with days where they had high training loads (i.e. ~1186 au; Lastella et al., 2020). This supports the notion that the sleep of youth athletes is susceptible to the training loads experienced. Furthermore, youth athletes who completed two sessions a day had a longer total sleep time compared with completing only one session or less (Gudmundsdottir, 2020). In addition, they also experienced a higher wake after sleep onset (WASO) when completing two sessions a day compared with just one session (Frytz et al., 2023). This potential contradiction between higher training load and number of sessions per day needs to be examined. There may be a point at which increased training loads combined with a suitable number of sessions could optimise sleep, although currently this relationship is not known.

Studies investigating the sleep of youth athletes have typically done so over short periods of time (i.e. < 14 days; Aloulou et al., 2021; Lastella et al., 2020; Steenekamp et al., 2021; Whitworth‐Turner et al., 2019). So, whilst these provide an indication of possible relationships between sleep and training in youth athletes, they do not provide any insight into longer‐term trends. They also do not take into consideration that the commitments youth athletes are facing are likely to vary week to week. For example, youth athletes often have differing academic and sport schedules. Some weeks they may have more training and games for their sport and therefore a higher training load, and other weeks they may have less. Similarly, their academic commitments may vary as some weeks they may have exams or heavy study periods. Therefore, these shorter study designs only provide a brief snapshot of the possible associations between training factors and sleep in these youth athletes.

Understanding the impact of training load, schedule and frequency on the sleep of youth athletes will assist practitioners in understanding how different aspects of training may be influencing sleep. Monitoring youth athletes over a longitudinal period of 8 weeks provides insight into the variety of different loads and schedules they experience and how these affected their sleep. As such, the aim of this study was to characterise the sleep of youth basketballers and investigate whether total training load, as well as the timing of training and games, affects the sleep of youth athletes over a longitudinal period. To understand the relationship between training and sleep, this study examined whether subjectively reported training load, time and frequency impact the objective sleep of youth athletes. It was expected that: (1) high training load; (2) later finishing time of training; and (3) a greater number of training sessions would have a negative impact on the sleep of youth athletes the following night. Furthermore, we assessed the effect of gender and intra‐individual variation on sleep variables to aid the discussion. It was hypothesised that female youth athletes would have better sleep compared with males (Aloulou et al., 2021), and that all youth athletes would have large intra‐individual variations in bed and sleep‐onset times and total sleep duration due to training commitments (Leduc et al., 2020).

## 2 METHODS

Forty youth athletes voluntarily participated in this study. Twenty‐six athletes (13 male and 13 female) completed a minimum of 50% of data collection (i.e. ≥ 28 days over 8 weeks) and were therefore included in the analysis (age: 14.22 ± 0.74 years [range 13–16 years]; height: 173.53 ± 13.71 cm; weight: 63.2 ± 11.92 kg). Participants included basketball athletes from a Victorian (Australia) school offering a high‐performance sporting program as part of their curriculum. Students are eligible for school entry based on their sporting excellence, and complete an integrated curriculum comprising of sporting sessions throughout the week (*n* = 4; average weekly training duration including inside and outside of school: 505 min) as well as their mandatory school subjects. This school was not a boarding institution, and the school day for participants started at 08:30 hours; however, it is possible that students could have had variable commute times to school each morning that were not collected as part of this study. Inclusion criteria required no athlete had any prior diagnosed sleep disorder. Athletes were asked to self‐report any sleep disorder diagnosis during screening, with none reported. Most participants (88%) consumed less than 1 caffeinated product per day, 8% consumed 1–2 per day, and 4% consumed 3 or more per day. Ethical approval was obtained prior to the beginning of the study from Deakin University [#2021‐225]. The study procedures were explained to participants and their parents/guardians before giving informed consent to participate.

The training load and sleep behaviours of participants were monitored over an 8‐week period during their school term. Baseline measures of subjective sleep quality, sleep hygiene and circadian phenotype were recorded. Activity monitors (Actigraph wGT3X‐BT) were worn nightly, and sleep and training load diaries were completed daily.

Three subjective sleep measures were collected at baseline. The Pittsburgh Sleep Quality Index (PSQI; Buysse et al., 1989) and Athlete Sleep Behaviour Questionnaire (ASBQ; Driller et al., 2018) were used to assess participants' sleep quality and sleep behaviours. The PSQI asks participants to record information about their habitual sleep (e.g. bedtime, sleep duration), with lower overall scores indicative of better sleep quality. Scores > 5 are indicative of “poor sleep quality”. The five questions rated by a bedroom partner were not used in the analysis due to the age of the participants. The ASBQ includes 18 items that are summed to create a global score ranging between 18 and 90. Question 4 was removed from the questionnaire prior to administration due to being associated with alcohol consumption. This was a request of the ethics committee due to the participants being under the age of 18 years. As a result, total scores could only range between 17 and 85. Therefore, based on original threshold values, the revised thresholds used were ≤ 34 = “good sleep behaviour” and ≥ 40 = “poor sleep behaviour”. The PSQI and ASBQ have been used previously in youth athlete populations (Aloulou et al., 2021; Ashby et al., 2024; Binti Abd Rahim et al., 2022). The Morningness–Eveningness Scale for Children (MESC) was used to obtain the chronotype of the participants (Carskadon et al., 1993) through a total of 10 questions surrounding morning or evening preference (e.g. Is it easy for you to get up in the morning?). Each answer is given a value between 1 and 5; these are then summed to create an overall score between 10 and 42. Scores between 10 and 20 indicate an evening type, scores between 28 and 42 indicate a morning type, and scores between 21 and 27 are classified as intermediate.

Objective sleep measures were obtained via an Actigraph wGT3X‐BT accelerometer (Actigraph, Pensacola, FL). These were configured with an epoch length of 60 s and sampling rate of 30 Hz. Participants were required to wear the actigraph on their non‐dominant wrist, as is commonly done in the literature (Aloulou et al., 2021; Patel et al., 2020; Steenekamp et al., 2021) and at night‐time before they went to sleep, and take them off in the morning when they woke up. At the conclusion of data collection, data from each actigraph were downloaded using the Actilife software (Actilife version 6.13.3, Actigraph, FL, USA). Having previously been validated in adolescent populations, the Sadeh algorithm was utilised to report on sleep measures (Sadeh et al., 1994).

Subjective measures of sleep were obtained through self‐reported sleep and training diaries completed on REDCap (Research Electronic Data Capture) via Deakin University (Harris et al., 2009; Harris et al., 2019). The sleep diary asked participants to document their bedtime and get‐up time, and to rate their subjective sleep quality on a five‐point Likert scale (1 = very good, 2 = good, 3 = average, 4 = poor, 5 = very poor), and perception of pre‐sleep fatigue and post‐awakening fatigue on a seven‐point Likert scale (1 = fully alert, wide awake, 7 = completely exhausted). Due to the potential for recall and social desirability bias, sleep diaries were used in conjunction with actigraphy (Halson, 2019). Sleep outcomes derived from actigraphy and sleep diaries were based on previous studies (Ashby et al., 2024; Gudmundsdottir, 2020; Lastella et al., 2020), and included:

- sleep latency (min; actigraphy): duration between bedtime and initial sleep onset;
- total sleep time (min; actigraphy): the total time spent asleep from initial sleep onset to get‐up time;
- time in bed (min; actigraphy): the total time in bed from bedtime to get‐up time;
- sleep onset (hours:min; actigraphy): the time‐of‐day an individual fell asleep after going to bed;
- WASO (min; actigraphy): the time between sleep onset and get‐up time spent awake;
- sleep efficiency (%; actigraphy): total sleep time expressed as percentage of time in bed;
- bedtime (hours:min; actigraphy): the time‐of‐day an individual began attempting to sleep;
- get‐up time (hours:min; actigraphy): the time‐of‐day an individual stopped attempting to sleep;
- subjective sleep quality (sleep diary): quality of the previous night's sleep on a scale of 0–5, with lower scores indicating better sleep quality;
- perception of pre‐sleep fatigue (sleep diary): fatigue rating prior to going to bed on a scale on 0–7, with higher scores indicating higher fatigue levels;
- post‐awakening fatigue (sleep diary): fatigue rating upon awakening on a scale of 0–7, with higher scores indicating higher fatigue levels.

Training load was monitored using session duration and sRPE. Athletes provided a rating of perceived exertion (RPE) and session duration (min) for each session, including both matches and training, that was completed, and this was multiplied to produce a sRPE (Foster, 1998). RPE is a measure where the athlete rates the session on a scale of 1 to 10 based on how difficult or easy the session was, with 1 being “nothing at all” and 10 being “very, very strong” (Borg, 1982). Athletes were trained on how to use the RPE scale and how to complete their responses. Details including time of training and games and type of trainings (e.g. court/field, strength, conditioning) were also recorded by the athletes. Athletes recorded this through their joint sleep and activity diary. sRPE has been previously used in studies as a valid and reliable measure to quantify training load in team sports (Impellizzeri et al., 2004). Additionally, sRPE has been shown as a suitable and valid measure in youth athlete populations (Scantlebury et al., 2018). Although RPE is traditionally completed within 30 min of training, in this study to maximise compliance, participants were asked to complete it at night. Completing RPE within 24 hr is still considered valid and reliable (Scantlebury et al., 2018), and by doing this it reduced the risk of participants being influenced by teammate responses. Training load was analysed as total daily training load (au), and was derived by summing the sRPE for every session completed on a single day (training and matches). Training duration was analysed as total daily duration (min), and was derived by summing the duration for every session completed on a single day (including both training and matches). Frequency of training sessions included number of daily training sessions (including both training and matches) completed per day.

Data were analysed through the Statistical Package for the Social Sciences (Version 25.0, IBM, Armonk, NY). Mean values are presented as mean (± standard deviation). Assumptions of normality were violated, and therefore data were not normally distributed. For all analyses, the *p*‐value was set at < 0.05. Daily sleep and training load trends were examined through descriptive analysis using mean and standard deviation. A chi‐square analysis compared the frequency of male and female athletes that did not achieve > 8 h sleep and > 85% sleep efficiency, as recommended for optimal health (Hirshkowitz et al., 2015; Ohayon et al., 2017). The intra‐individual variation for the sleep variables of latency, efficiency, total time in bed, total sleep time, WASO, bedtime, sleep‐onset time and wake‐up time were calculated. Generalised estimating equations (GEE) were used to assess the relationship between training load, training time, training frequency and each sleep variable. For each GEE, subject effect was each participant, within subject effect was week number and the sleep variable included, the dependent variable. The factors were gender, number of training sessions per day, and time‐of‐day at completion of final training session (< 09:00 hours, 09:00 hours–12:00 hours, 12:00 hours–15:00 hours, 15:00 hours–18:00 hours, 18:00 hours–20:30 hours, > 20:30 hours). These categories were selected to account for training sessions completed before school, during school and after school, which was broken down into straight after school (15:00 hours–18:00 hours), evening (18:00 hours–20:30 hours) or late (after 20:30 hours). Two separate models (one for training load and one for training duration) were run to examine how training factors were associated with each sleep variable. Duration and load were not included in the same model because these variables were strongly correlated (*r* = 0.87). Both models included training session time, frequency, gender and the sleep variable, alongside either training duration or training load. The data included values of zero for days when no training was completed; however, GEE automatically remove zero values. Descriptive statistics do include zeros in the calculation.

## 3 RESULTS

**FIGURE 1:**
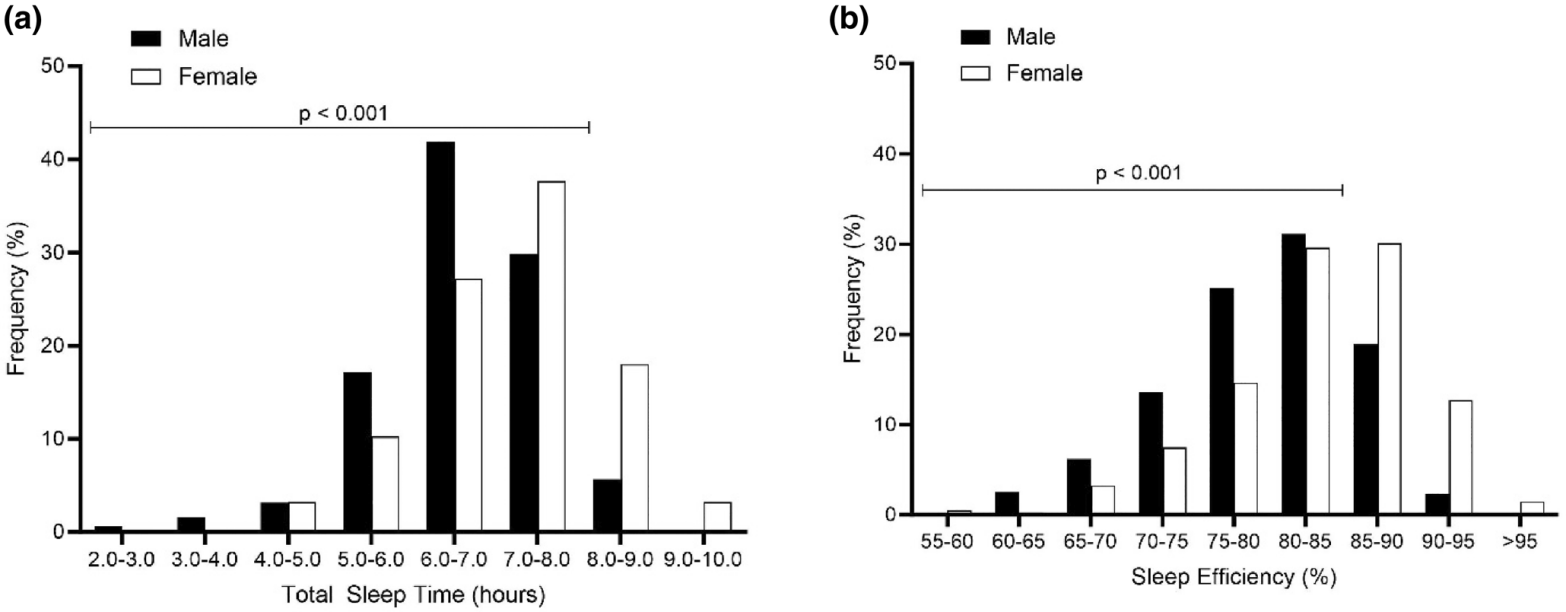
Frequency distributions of participants (a) total sleep time and (b) sleep efficiency. Data are presented as a percentage of total male and female participants. Compared with females, males more often averaged < 8 hr of sleep each night (*χ* ^2^ = 47.78, *p* < 0.001). Compared with females, males more often averaged less than 85% sleep efficiency (*χ* ^2^ = 55.81, *p* < 0.001).

Sleep outcomes and gender differences are presented in Table 1. Participants averaged 6.85 hr of sleep each night, and had an average sleep‐onset time of 23:08 hours. Significant differences were observed between males and females for all sleep variables, apart from bedtime. Total sleep time of males was 0.59 hr (*p* < 0.05) less than females. Figure 1 presents the frequency distribution between male and female basketballers in respect to total sleep time and sleep efficiency. For most (79%) of the nights included in the study, females averaged less than 8 hr of sleep each night compared with 94% of the nights for males (*χ* ^2^ = 47.78, *p* < 0.001). Females averaged less than 85% efficiency on 56% of the nights studied compared with 79% of the nights for males (*χ* ^2^ = 55.81, *p* < 0.001). The average PSQI value was 6 (± 4), with 11 participants having PSQI values above 5, which is the established threshold for “reduced sleep quality” (Buysse et al., 1989). The average ASBQ value across participants was 37 (± 7), with 36% having scores ≥ 40, which is considered “poor” based on adjusted thresholds. Most participants reported a morning chronotype (*n* = 23), whilst two were considered intermediate and one did not provide chronotype data. Intra‐individual variation in sleep variables are shown in Table 1. Participants had almost an hour (0.84 hr) of intra‐individual variation for total sleep time, and 1.03 hr and 1.02 hr for sleep‐onset time and wake‐up time, respectively.

**TABLE 1:** Sleep variables including subjective sleep responses and objective values from an actigraph and intra‐individual variations

	Overall			Intra‐individual variation		
Variable	Females	Males	Overall	Females	Males	Overall
Subjective sleep quality (au)	2.30 (0.98)	2.04 (0.96)^b^	2.15 (0.99)	–	–	–
Fatigue rating upon awakening (au)	3.69 (1.21)	3.44 (1.41)^b^	3.57 (1.35)	–	–	–
Fatigue rating right now before bed (au)	3.93 (1.22)	3.78 (1.52)^b^	3.82 (1.38)	–	–	–
Total time in bed (hr)^a^	8.63 (1.05)	8.33 (1.12)^b^	8.42 (1.08)	0.94 (0.21)	0.89 (0.39)	0.92 (0.31)
Total sleep time (hr)^a^	7.20 (1.11)	6.61 (1.03)^b^	6.85 (1.08)	0.89 (0.20)	0.79 (0.29)	0.84 (0.25)
Latency (min)^a^	13.93 (12.12)	17.29 (17.58)^b^	15.92 (15.58)	10.05 (4.44)	12.61 (8.16)	11.33 (6.57)
Efficiency (%)^a^	83.31 (6.64)	79.29 (6.75)^b^	81.24 (6.90)	5.38 (1.42)	5.39 (1.22)	5.39 (1.29)
WASO (min)^a^	71.63 (31.67)	86.27 (33.11)^b^	74.48 (32.98)	26.00 (5.59)	27.64 (7.01)	26.82 (6.27)
Bedtime (HH:MM)^a^	22:45 (1.16)	22:57 (1.25)	22:52 (1.22)	1.07 (0.48)	1.06 (0.51)	1.06 (0.49)
Sleep‐onset time (HH:MM)^a^	22:59 (1.16)	23:14 (1.29)^b^	23:08 (1.22)	1.02 (0.37)	1.04 (0.45)	1.03 (0.40)
Wake‐up time (HH:MM)^a^	7:22 (1.07)	7:17 (1.14)^b^	7:18 (1.10)	0.98 (0.29)	1.07 (0.36)	1.02 (0.33)

Results are presented as mean (standard deviation).

au, arbitrary units; WASO, wake after sleep onset.

^a^ Measures were taken from an Actigraph. All time variables are using 24‐hr time.

^b^ Significantly different from females.

A total of 309 matches and 868 training sessions were assessed across the duration of the study. Data were collected during weekdays and weekends. A total of 844 sessions were recorded on weekdays and 333 on weekends. The average training start time of participants was 14:54 hours (± 4.12 hr) and average finish time was 16:26 hours (± 4.01 hr). All the participants experienced at least one training finish (later than 20:30 hours) during the study. Across the duration of the study, there were a total of 199 sessions that had their end time of latest training after 20:30 hours (before 09:00 hours: 7; 09:00 hours–12:00 hours: 81; 12:00 hours–15:00 hours: 157; 15:00 hours–18:00 hours: 130; 18:00 hours–20:30 hours: 289). The breakdown of the latest time of training on weekdays is as follows; before 09:00 hours: 0.96%; 09:00 hours–12:00 hours: 10.06%; 12:00 hours–15:00 hours: 23.64%; 15:00 hours–18:00 hours: 14.38%; 18:00 hours–20:30 hours: 37.86%; after 20:30 hours: 13.10%. The breakdown of the latest time of training on weekends is as follows; before 09:00 hours: 0.42%; 09:00 hours–12:00 hours: 7.59%; 12:00 hours–15:00 hours: 3.80%; 15:00 hours–18:00 hours: 16.88%; 18:00 hours–20:30 hours: 21.94%; after 20:30 hours: 49.37%. The average duration of individual training sessions was 125.03 min (± 77.47 min), and they completed zero sessions on 22.25% of days, one session on 53.24% of days, two sessions on 20.72% of days, and three sessions on 3.79% of days. The average training duration on weekdays was 130.54 ± 78.82 min, while on weekends it was 110.51 ± 71.63 min. Their average daily load was 587.21 au (± 642.51 au) when rest days were included. Males had an average daily load including rest days of 669.17 au (± 719.49 au) and females 478.35 au (± 453.84 au). When rest days were excluded, the daily load of participants was 749.57 au (± 608.42 au) ales 864.64 au [± 706.99 au], females 612.24 au [± 426.13 au]). The average training load on weekdays was 758.95 ± 607.72 au and 724.81 ± 608.31 au on weekends.

Relationships between training time, duration, frequency and sleep are presented in Table 2. For total sleep time, if training ended between 12:00 hours and 15:00 hours (*p* < 0.001) or 15:00 hours–18:00 hours (*p* = 0.002), this resulted in 0.38 hr and 0.42 hr more total sleep time, respectively, than when training ended after 20:30 hours. There were no significant differences in total sleep time for trainings that ended before 09:00 hours, between 09:00 hours–12:00 hours, and between 18:00 hours–20:30 hours when compared with trainings that ended after 20:30 hours. For bedtime, if training ended between 09:00 hours and 12:00 hours (*p* < 0.001), or 12:00 hours–15:00 hours (*p* < 0.001), or 15:00 hours–18:00 hours (*p* < 0.001), or 18:00 hours–20:30 hours (*p* < 0.001), their bedtime was 1.09 hr, 1.28 hr, 1.22 hr and 1.26 hr earlier, respectively, when compared with training that ended after 20:30 hours. For sleep‐onset time, if training ended before 09:00 hours (*p* = 0.04), or between 09:00 hours and 12:00 hours (*p* < 0.001), or 12:00 hours–15:00 hours (*p* < 0.001), or 15:00 hours–18:00 hours (*p* < 0.001), or 18:00 hours–20:30 hours (*p* < 0.001), their sleep‐onset time was 1.33 hr, 1.06 hr, 1.24 hr, 1.23 hr and 1.24 hr earlier, respectively, when compared with a training that ended after 20:30 hours. For wake‐up time, if training ended before 09:00 hours (*p* = 0.004), or between 09:00 hours–12:00 hours (*p* < 0.001), or 12:00 hours–15:00 hours (*p* < 0.001), or 15:00 hours–18:00 hours (*p* < 0.001), or 18:00 hours–20:30 hours (*p* < 0.001), their wake‐up time the following day was 0.93 hr, 1.06 hr, 0.95 hr, 0.81 hr and 0.92 hr earlier, respectively, when compared with training that ended after 20:30 hours. No significant effects were found for sleep latency, sleep efficiency and WASO in relation to training time. However, a 10‐min increase in total daily duration resulted in a 0.03 hr decrease in total sleep time (*p* = 0.003) and 0.2% decrease in sleep efficiency (*p* = 0.002), and a 1.10 min increase in WASO (*p* = 0.01). For total sleep time, if participants completed two training sessions in a day, this resulted in 0.33 hr more total sleep time compared with one training session. For latency, if participants completed three training sessions in a day, their latency was 4.94 min less when compared with one training session. For sleep efficiency, if participants completed two or three sessions, their sleep efficiency was 2.14% and 3.03% higher compared with one session. For WASO, if participants completed two sessions, their WASO was 7.67 min less than if they completed one session. The results of the GEE examining training time, frequency and sRPE on sleep are presented in Table S1.

**TABLE 2:** Results of GEE investigating the impact of training time, total sessions and daily duration on sleep variables

	Total sleep time	Latency	Efficiency	WASO	Bedtime	Onset time	Wake‐up time
	β (95% CI)	β (95% CI)	β (95% CI)	β (95% CI)	β (95% CI)	β (95% CI)	β (95% CI)
	*p*‐Value	*p*‐Value	*p*‐Value	*p*‐Value	*p*‐Value	*p*‐Value	*p*‐Value
End time of last training (before 09:00 hours)	0.47	−3.79	1.95	−4.17	−1.26	−1.33	−0.93
	(−0.48, 1.43)	(−10.55, 2.96)	(−2.76, 6.65)	(−26.29, 17.95)	(−2.56, 0.03)	(−2.62, −0.05)	(−1.55, −0.30)
	0.33	0.27	0.42	0.71	0.05	0.04*	0.004*
End time of last training (09:00 hours–12:00 hours)	−0.03	1.13	−0.62	2.71	−1.09	−1.06	−1.06
	(−0.34, 0.28)	(−4.58, 6.84)	(−2.38, 1.14)	(−7.67, 13.08)	(−1.57, −0.62)	(−1.49, −0.62)	(−1.49, −0.62)
	0.86	0.70	0.49	0.61	< 0.001*	< 0.001*	< 0.001*
End time of last training (12:00 hours–15:00 hours)	0.38	2.41	1.09	−4.69	−1.28	−1.24	−0.95
	(0.16, 0.60)	(−2.58, 7.40)	(−0.58, 2.74)	(−15.64, 6.26)	(−1.72, −0.85)	(−1.68, −0.81)	(−1.31, −0.58)
	< 0.001*	0.34	0.20	0.40	< 0.001*	< 0.001*	< 0.001*
End time of last training (15:00 hours–18:00 hours)	0.42	−0.73	0.96	0.15	−1.22	−1.23	−0.81
	(0.16, 0.60)	(−4.36, 3.90)	(−1.09, 3.00)	(−11.09, 11.39)	(−1.62, −0.81)	(−1.61, −0.85)	(−1.17, −0.45)
	0.002*	0.69	0.36	0.92	< 0.001*	< 0.001*	< 0.001*
End time of last training (18:00 hours–20:30 hours)	0.27	1.48	−0.26	3.08	−1.26	−1.24	−0.92
	(−0.02, 0.55)	(−1.25, 4.21)	(−1.65, 1.14)	(−3.60, 9.77)	(−1.58, −0.95)	(−1.54, −0.94)	(−1.25, −0.59)
	0.07	0.36	0.72	0.37	< 0.001*	< 0.001*	< 0.001*
End time of last training (after 20:30 hours)							
Total sessions = 3	0.29	−4.94	3.03	−8.93	−0.05	−0.13	0.003
	(−0.06, 0.63)	(−9.45, −0.43)	(0.12, 5.94)	(−24.25, 6.39)	(−0.58, 0.47)	(−0.66, 0.40)	(−0.49, 0.50)
	0.10	0.03*	0.04*	0.25	0.84	0.62	0.99
Total sessions = 2	0.33	−1.45	2.14	−7.67	−0.13	−0.16	0.04
	(0.08, 0.58)	(−4.72, 1.82)	(−0.73, 3.55)	(−14.13, −1.21)	(−0.49, 0.22)	(−0.52, 0.21)	(−0.22, 0.31)
	0.01*	0.39	0.003*	0.02*	0.46	0.40	0.75
Total sessions = 1							
Total daily duration (× 10 min)	−0.03	0.02	−0.20	1.10	< −0.001	< −0.001	−0.01
	−0.04, −0.01)	(−0.20, 0.20)	(−0.40, −0.10)	(0.30, 1.90)	(−0.20, 0.20)	(−0.02, 0.02)	(−0.02, 0.01)
	0.003*	0.86	0.002*	0.01*	0.99	0.99	0.41

Results are compared with each blank row. For example, each end time of last training is compared with the end time of last training after 20:30 hours. This is the same for total sessions.

CI, confidence interval; WASO, wake after sleep onset.

* Denotes statistical significant of *p* < 0.05.

## 4 DISCUSSION

This study investigated the impact of timing, load and frequency of training on the sleep of youth basketball athletes. When training finished later than 20:30 hours at night, youth athletes had significantly less total sleep time than when training finished prior to 20:30 hours. Increases in daily training duration resulted in decreases in total sleep time and sleep efficiency and increases in WASO. Youth athletes who completed two training sessions had a longer total sleep time than when they completed one session, and sleep efficiency was improved for athletes who trained twice or more each day.

In the current study, late night scheduling of training sessions had a significant impact on the sleep of youth basketballers. If they finished their training between 12:00 hours and 18:00 hours, they had over 20 min more sleep than when finishing training after 20:30 hours. In addition to total sleep time, youth athletes were getting to bed an hour earlier if training finished between 09:00 hours and 20:30 hours than when finishing training after 20:30 hours. This is particularly interesting, as most of the participants (92%, *n* = 23) reported they were a morning chronotype, suggesting participants may want to go to bed earlier but are restricted by their training schedule. Furthermore, youth athletes were found to wake up close to an hour later if their training finished after 20:30 hours the preceding night. While youth athletes may not have the flexibility in deciding their wake‐up time during the week (due to school starting times), in the current study 56% of the training sessions finishing after 20:30 hours occurred on Friday night. This may explain why athletes were able to sleep for the additional hour if training finished after 20:30 hours. Previous research has shown that youth academy footballers went to sleep significantly later after evening training sessions when compared with before morning sessions (Brown et al., 2020). Additionally, the median sleep duration of youth swimming and rowing athletes was significantly shorter on nights before morning trainings compared with when they had the day off (Steenekamp et al., 2021). This emphasises the impact of the training schedules of youth athletes on when they can go to sleep and therefore how much sleep they can obtain. Ideally, training sessions for youth athletes should be earlier in the evening to increase opportunities for improved sleep behaviour. However, this may be difficult to implement due to school demands (i.e. finishing time) and reliance on parents for transportation to training. Future research could seek to trial afternoon and early evening training sessions (i.e. not evenings) for youth athletes, and investigate whether this leads to improved sleep habits.

In the present research, for every 10‐min increase in daily training duration there was a 1 min 48 s decrease in total sleep time, 0.2% decrease in sleep efficiency, and a 1.1‐min increase in WASO. Prior studies have demonstrated that higher training loads resulted in lower total sleep time (Aloulou et al., 2021; Dumortier et al., 2018), higher sleep fragmentation (Aloulou et al., 2021) and lower sleep efficiency (Lastella et al., 2020). In addition, previous research has shown that increases in muscle damage and inflammation are associated with poorer sleep and more specifically sleep onset (Fox et al., 2020). Therefore, it could be hypothesised that an increase in training duration in youth athletes could be linked to an increase in muscle damage and consequently poorer sleep. Exercise can also lead to an increased sympathetic activation, which is not beneficial for sleep (Yamanaka et al., 2015).

Youth athletes who completed two sessions experienced an increase in total sleep time compared with those who only completed one session, but this was not observed when they completed three sessions. Sleep efficiency was improved for athletes who completed two or more training sessions per day. This suggests that the sleep of youth athletes is improved with two sessions in a day compared with one or three. However, our results also showed that a greater total duration of training per day negatively impacted sleep. To date, only one study in youth athletes has investigated both training duration and training frequency on sleep, and it similarly found that two training sessions resulted in longer total sleep duration compared with just one session (Frytz et al., 2023). However, in that study, all training sessions were 90 min, therefore this limits the ability to account for variations in session durations in relation to session frequency. It is common for training sessions to vary in length, therefore, this current study highlights a potential interaction between session duration and session frequency. Future research should seek to identify whether training adaptations can be optimised with shorter, more frequent training sessions (i.e. two shorter sessions) that also enable improved sleep behaviours and opportunities to maximise sleep.

Compared with female youth basketballers, males slept on average 35 min less each night. This finding is similar to a previous study that compared nine different youth sports, and reported that males slept 32.9 min less than females (Aloulou et al., 2021). Conversely, another study observed no differences between the total sleep time of male and female youth athletes (Skein et al., 2019). Sleep efficiency was lower than the recommended level (85%) for both male and female youth athletes (Ohayon et al., 2017). These findings, alongside 11 youth athletes having PSQI values above 5, the established threshold for “sleep problems”, suggest that they are not exhibiting good sleep habits as well as not meeting the recommendations for good sleep (Buysse et al., 1989). Low sleep time, poor sleep efficiency and subjective “sleep problems” could put youth athletes at risk of development delays (Beebe, 2011), and negative impacts on cognitive function and wellbeing (Louca & Short, 2014).

A concerning finding of this study is the limited sleep that youth athletes were getting, which was considerably less than the recommend 8–10 hr of sleep that is required for optimal functioning and good health in this age group (Beebe, 2011; Hirshkowitz et al., 2015). Moreover, none of the participants achieved the higher end of the recommendation, 10 hr. Participants averaged 6.85 hr of total sleep, and the average sleep onset time was 23:08 hours. For the majority of the nights studied for male (79%) and female participants (56%), they averaged less than the recommended 85% sleep efficiency. Additionally, participants exhibited large intra‐individual variations in bedtime (1.066 hr) and total sleep time (0.84 hr). Although the current study observed low total sleep durations, it is consistent with other studies with youth athletes, whereby participants have self‐reported an average of 7 hr each night (Suppiah et al., 2021) and objective measures have recorded an average of 7.1 hr (Aloulou et al., 2021). One possible explanation for the low total sleep time in this cohort could be limited sleep opportunity (i.e. late bedtime and early wake‐up time [to attend school]). Although most participants self‐reported being “morning” chronotypes, mean bedtime during the data collection period was nonetheless late (22:52 hours). Poor sleep hygiene and/or evening training sessions may explain these findings. For example, ASBQ results revealed 36% of participants had “poor” sleep hygiene, which may have led to delayed bedtimes for these participants. Further, 57% of training sessions finished after 18:00 hours, and thus bedtimes may have been delayed due to delayed evening routines (i.e. travel home, later evening meals) or physiological arousal resulting from intense exercise (Yamanaka et al., 2015).

An important finding of this study was the large intra‐individual variation in bedtime, sleep‐onset time and wake‐up time. Youth athletes in this study had a mean intra‐individual variation of their bedtime of 1 hr (1.06). This is similar to a study in junior rugby league athletes who had an intra‐individual variation of greater than 1 hr in their bedtime, onset time and wake‐up time (Caia et al., 2017). This intra‐individual variation in their sleep times may be unavoidable for youth athletes due to their schedules. Previously, research in adult athletes has showed lower intra‐individual variation, and this could be due to consistent scheduling at an elite level (Caia et al., 2017). Unfortunately, youth athletes do not always get this same opportunity, and may have matches or trainings scheduled at different times across the week, making it nearly impossible to obtain consistent bedtimes and wake‐up times. Having regular bedtimes is an important aspect of healthy sleep hygiene (Bird, 2013). Previously, when youth tennis players consistently adhered to an early bedtime (21:30 hours) and adopted other sleep hygiene behaviours, total sleep time increased (Lever et al., 2021). Therefore, awareness of good sleep hygiene is extremely important for youth athletes; and importantly, as it may not be possible in many cases to go to bed early (i.e. following evening training) or to wake later (i.e. due to school start times), it is critical youth at least understand the value of consistent sleep–wake patterns for optimising sleep. Additionally, as sleep leads to performance improvements, this should be an important consideration for practitioners who are scheduling training and match activities to promote positive sleep behaviours in youth athletes.

A limitation of this study was that 50% completion was the minimum for data to be included in this study. Although this could be considered a low completion rate, this was decided upon to allow the most participants to be included in the study whilst also ensuring enough data were available. The use of a GEE in the data analysis allowed for the incorporation of missing data. Another limitation is that only youth basketball athletes were used in this study, therefore the results may not be generalisable to the wider youth athlete population. Future research could seek to investigate training and sleep in additional youth sport athletes as this may present a broader variety of training schedules and load. In addition, this study was conducted during a school term, and bed and wake‐up times may vary in school holidays. Future research could investigate bed and wake times of youth athletes during school holidays, and the subsequent relationship with their training schedules. In addition, the comparison between weekend and weekday training load, frequency and timing could also be explored. Furthermore, as the study was observational in nature, this limits the ability to make assumptions around causality. It is important to note that in the analysis, training sessions and matches were combined and not analysed separately. This may be considered a limitation as matches may have a different effect on athletes compared with trainings. However, this was decided to simplify the assessment of overall training load and its impact on sleep, in addition to providing a larger sample size for analysis.

## 5 CONCLUSION

The sleep of youth basketballers is being impacted by their total training load and the timing of training and/or games. A reduction in total sleep time and a later bedtime and sleep‐onset time were exhibited when youth athletes finished training after 20:30 hours. In addition, increases in training duration were associated with decreases in total sleep time and sleep efficiency. Youth athletes who completed two training sessions in a day had more sleep than those who only completed one. Across the study their average total sleep time was 6.85 hr. Most male (94%) and female (79%) youth athletes did not meet the minimum recommended 8 hr of sleep for their age group. This can be detrimental for their growth and development as well as not providing youth athletes enough opportunity to recover from their sporting commitments.

## 6 PRACTICAL IMPLICATIONS

The findings of the current study extend previous work investigating the impact of training on the sleep of youth athletes over an 8‐week period (Aloulou et al., 2021; Lastella et al., 2020). These data should be used by coaches and sports administrators to advocate for the need to finish training sessions for youth athletes by 20:30 hours to enable them to achieve a minimum 8 hr sleep. This will assist in promoting appropriate bedtimes and improve the opportunity for an increased total sleep time. Coaches should also be aware of the impact of training duration on the sleep of youth athletes, and plan training sessions accordingly. Additionally, youth athletes should be educated on the importance of good sleep hygiene, consistent bedtimes and wake‐up times. Finally, youth athletes need their sporting organisations to understand and acknowledge the impact that late games and training times as well as training duration have on their sleep.

## AUTHOR CONTRIBUTIONS

**Tanisha Tate:** Conceptualization; investigation; methodology; writing – original draft; writing – review and editing; project administration; formal analysis; data curation. **Spencer Roberts:** Conceptualization; investigation; methodology; writing – review and editing; formal analysis; project administration; data curation; supervision. **Luana C. Main:** Conceptualization; investigation; writing – review and editing; methodology; formal analysis; project administration; supervision; data curation. **Lyndell Bruce:** Conceptualization; investigation; writing – review and editing; methodology; formal analysis; project administration; data curation; supervision.

## CONFLICT OF INTEREST STATEMENT

The authors declare no conflicts.

## Supporting information


**TABLE S1.** Results of GEE investigating the impact of training time, total sessions and daily load on sleep variables.

## Data Availability

The data that support the findings of this study are available on request from the corresponding author. The data are not publicly available due to privacy or ethical restrictions.
