# Supplementary material for: The influence of training load and schedule on youth athletes' sleep
Source: J Sleep Res. 2025 Feb 17;34(6):e70013. doi: 10.1111/jsr.70013 (PMC12592842; doi:10.1111/jsr.70013)
Supplement: Supplementary file 1 — TABLE S1. Results of GEE investigating the impact of training time, total sessions and daily load on sleep variables. [file JSR-34-e70013-s001.docx]

|  | **Total Sleep Time** | **Latency** | **Efficiency** | **WASO** | **Bedtime** | **Onset time** | **Wake up time** |
| --- | --- | --- | --- | --- | --- | --- | --- |
|  | **β (95% CI)**  **p-value** | **β (95% CI)**  **p-value** | **β (95% CI)**  **p-value** | **β (95% CI)**  **p-value** | **β (95% CI)**  **p-value** | **β (95% CI)**  **p-value** | **β (95% CI)**  **p-value** |
| End time of last training  (Before 9am) | .52  (-.45, 1.48)  .29 | -4.28  (-11.07, 2.51)  .22 | 2.27  (-2.64, 7.18)  .37 | -5.05  (-27.55, 17.45)  .66 | -1.26  (-2.55, .02)  .05 | -1.34  (-2.61, -.07)  .04* | -.90  (-1.51, -.29)  .004* |
| End time of last training  (9am-12pm) | -.04  (-.35, .27)  .79 | 1.27  (-4.53, 7.07)  .67 | -.72  (-2.49, 1.05)  .42 | 3.00  (-7.40, 13.39)  .57 | -1.10  (-1.57, -.63)  <.001* | -1.06  (-1.49, -.63)  <.001* | -1.07  (-1.51, -.62)  <.001* |
| End time of last training  (12pm-3pm) | .35  (.12, .58)  .003* | 2.84  (-2.03, 7.72)  .25 | .93  (-.73, 2.59)  .27 | -4.56  (-15.52, 6.39)  .41 | -1.29  (-1.74, -.84)  <.001* | -1.24  (-1.70, -.79)  <.001* | -.97  (-1.35, -.58)  <.001* |
| End time of last training  (3pm-6pm) | .39  (.12, .66)  .01* | .02  (-3.64, 3.68)  .99 | .89  (-1.11, 2.88)  .39 | -.56  (-11.51, 10.39)  .92 | -1.23  (1.65, -.80)  <.001* | -1.23  (-1.63, -.82)  <.001* | -.84  (-1.22, -.46)  <.001* |
| End time of last training  (6pm-8:30pm) | .27  (-.02, .57)  .07 | 1.35  (-1.49, 4.18)  .35 | -.20  (-1.63, 1.22)  .78 | 3.02  (-3.58, 9.62)  .37 | -1.26  (-1.58, -.94)  <.001* | -1.24  (-1.54, -.94)  <.001* | -.92  (-1.25, -.59)  <.001* |
| End time of last training  (After 8:30pm) |  |  |  |  |  |  |  |
| Total sessions = 3 | -.12  (-.44, .20)  .46 | -3.69  (-8.56, 1.19)  .14 | 1.75  (-.84, 4.35)  .19 | -4.57  (-17.20, 8.06)  .48 | -.07  (-.52, .39)  .78 | -.12  (-.58, .33)  .59 | -.08  (-.57, .41)  .75 |
| Total sessions = 2 | .19  (-.04, .42)  .10 | -.34  (-3.37, 2.69)  .83 | 1.11  (-.33, 2.56)  .13 | -4.28  (-10.53, 1.98)  .18 | -.14  (-.42, .14)  .32 | -.15  (-.43, .14)  .32 | -.03  (-.27, .21)  .82 |
| Total sessions = 1 |  |  |  |  |  |  |  |
| Total Daily Load (x 100au) | <.001  (<.001, <.001)  .09 | -.20  (-.60, .20)  .28 | -.20  (-.04, <.001)  .03* | 1.00  (.50, 2.00)  .002* | <.001  (<.001, <.001)  .81 | <.001  (<.001, <.001)  .84 | <.001  (<.001, <.001)  .82 |

**Supplementary Table 1. Results of generalised estimating equation investigating the impact of training time, total sessions and daily load on sleep variables.**

Note: Results are compared to each blank row. For example each end time of last training is compare to the end time of last training after 8:30pm. This is the same for total sessions. * denotes statistical significant of p < .05.
